# Internal Cholinergic Regulation of Learning and Recall in a Model of Olfactory Processing

**DOI:** 10.3389/fncel.2016.00256

**Published:** 2016-11-08

**Authors:** Licurgo de Almeida, Marco Idiart, Owen Dean, Sasha Devore, David M. Smith, Christiane Linster

**Affiliations:** ^1^Computational Physiology Lab, Department of Neurobiology and Behavior, Cornell UniversityIthaca, NY, USA; ^2^Physics InstituteFederal University of Rio Grande do Sul (UFRGS)Porto Alegre, Brazil; ^3^Department of Psychology, Cornell UniversityIthaca, NY, USA

**Keywords:** acetylcholine, olfactory bulb, olfactory cortex, regulation, learning and memory

## Abstract

In the olfactory system, cholinergic modulation has been associated with contrast modulation and changes in receptive fields in the olfactory bulb, as well the learning of odor associations in olfactory cortex. Computational modeling and behavioral studies suggest that cholinergic modulation could improve sensory processing and learning while preventing pro-active interference when task demands are high. However, how sensory inputs and/or learning regulate incoming modulation has not yet been elucidated. We here use a computational model of the olfactory bulb, piriform cortex (PC) and horizontal limb of the diagonal band of Broca (HDB) to explore how olfactory learning could regulate cholinergic inputs to the system in a closed feedback loop. In our model, the novelty of an odor is reflected in firing rates and sparseness of cortical neurons in response to that odor and these firing rates can directly regulate learning in the system by modifying cholinergic inputs to the system. In the model, cholinergic neurons reduce their firing in response to familiar odors—reducing plasticity in the PC, but increase their firing in response to novel odor—increasing PC plasticity. Recordings from HDB neurons in awake behaving rats reflect predictions from the model by showing that a subset of neurons decrease their firing as an odor becomes familiar.

## Introduction

Cholinergic modulation has been associated with contrast modulation and changes in receptive fields in a number of sensory processing areas including olfaction (Metherate and Weinberger, 1989; Linster and Cleland, 2002; Chaudhury et al., 2009; Alitto and Dan, 2012; Ma and Luo, 2012; de Almeida et al., 2013; Pinto et al., 2013). Computational modeling and behavioral studies suggest that cholinergic modulation could improve sensory processing and learning when task demands are high, however, how sensory inputs and or learning regulate incoming modulation has not yet been elucidated. Recent recordings of modulatory activity in the horizontal limb of the diagonal band of Broca show that activity in this nucleus is strongly modulated during olfactory tasks and can be related to task difficulty and degree of task acquisition of the task (Devore et al., 2016).

We here use a computational model of the olfactory bulb (OB), piriform cortex (PC) and horizontal limb of the diagonal band of Broca (HDB) to explore how olfactory learning can regulate cholinergic inputs to the system in a closed feedback loop. We and others have previously proposed that in computational models, cholinergic modulation in the OB can regulate odor representations and neural synchrony, thus changing odor inputs to the cortex (de Almeida et al., 2013; Li and Cleland, 2013; Devore et al., 2014). In piriform cortex, cholinergic modulation is thought to aid associative memory function by reducing interference between learned information and by increasing neural excitability and synaptic plasticity (Hasselmo et al., 1992; Patil et al., 1998; De Rosa and Hasselmo, 2000; De Rosa et al., 2001). We here show computationally that if the quality of learning in the PC directly regulates levels of ACh in OB and PC, the system can switch between encoding of new information and recall of previously learned information in a continuous and self-regulated manner.

In the olfactory system, ACh modulates neuronal groups from both olfactory bulb (OB) and piriform cortex (PC). In the OB, Ach has been shown to modulate principal cells and different classes of interneurons via both nicotinic and muscarinic receptors (Ravel et al., 1990; Castillo et al., 1999; Crespo et al., 2000; Pressler et al., 2007; D'souza and Vijayaraghavan, 2012; Ma and Luo, 2012; D'souza et al., 2013; Rothermel et al., 2014; Liu et al., 2015; Smith et al., 2015; Bendahmane et al., 2016). From these data, the net functional effect of ACh inputs to the OB can be constructed as enhancing mitral cell selectivity to odorants through increased inhibition and filtering of low amplitude inputs in concert with increased excitability in response to selective odorants; this idea is supported by behavioral and electrophysiological data (Elaagouby et al., 1991; Linster et al., 2001; Wilson et al., 2004; Mandairon et al., 2006; Devore et al., 2014). In the PC, ACh has been shown to have effects on principal cell and interneuron excitability and afterhyperpolarization, excitatory and inhibitory synaptic transmission as well as synaptic plasticity (Williams and Constanti, 1988; Tseng and Haberly, 1989; Hasselmo et al., 1992; Barkai and Hasselmo, 1994; Barkai et al., 1994; Hasselmo and Barkai, 1995; Hasselmo and Cekic, 1996; Patil et al., 1998; Patil and Hasselmo, 1999; Haberly, 2001). Based on computational investigations (reviewed in Hasselmo and Giocomo, 2006), cholinergic modulation in PC improves associative memory function by globally increasing excitability and plasticity and specifically suppressing previously encoded information during learning (Linster et al., 2003). Both OB and PC receive (presumably common) cholinergic inputs from the medial pre-optic area and particularly from the horizontal limb of the diagonal band of Broca (HDB) (Brashear et al., 1986; Záborszky et al., 1986) and electrical stimulation of axons coming out of the OB or principal cells in the PC results in firing rate modulation of neurons in the HDB (Linster and Hasselmo, 2000), suggesting the possible existence of a feedback loop between the HDB and olfactory structures. Our previous work has shown that concerted cholinergic modulation in OB and PC improve cortical learning and associative memory function (de Almeida et al., 2013; Devore et al., 2014), due to improved signal-to-noise ratio and synchronization in OB (which results in better cortical read-out) and increased excitability and plasticity in PC. Pyramidal cell networks trained using modulated inputs from the bulb exhibit more robust learning, with stronger neuronal activation and sparser cortical representations of odorants. These more robust memories are, at the same time, more distinct from each other and more resistant to noise than those trained with unmodulated bulbar inputs.

In previous computational models of cholinergic modulation, levels of ACh were regulated *ad hoc* (Barkai et al., 1994; Hasselmo and Cekic, 1996; Linster and Gervais, 1996; Linster and Hasselmo, 1997; Linster and Cleland, 2002; Linster et al., 2007; de Almeida et al., 2013) and possible mechanisms regulating the level of ACh were not explored. While these may be dependent on extrinsic factors such as task demands and attentional state for example, levels of ACH could also be regulated directly by activity in the olfactory pathway. We here show that in our existing a model of olfactory processing, cholinergic activity can be regulated by inputs from the PC in such a manner as to smoothly modulate odor responses in the OB and cortex. The network transitions between learning and recall modes automatically, based on the quality of associative memory formed in the PC. Recordings in the horizontal limb of the HDB in awake behaving rats in a simple odor familiarization task showed that as rats become more familiar with an odor, baseline neural activity in a small fraction of cells follows the predictions from our computational model.

## Methods

### Network architecture

**Figure 1:**
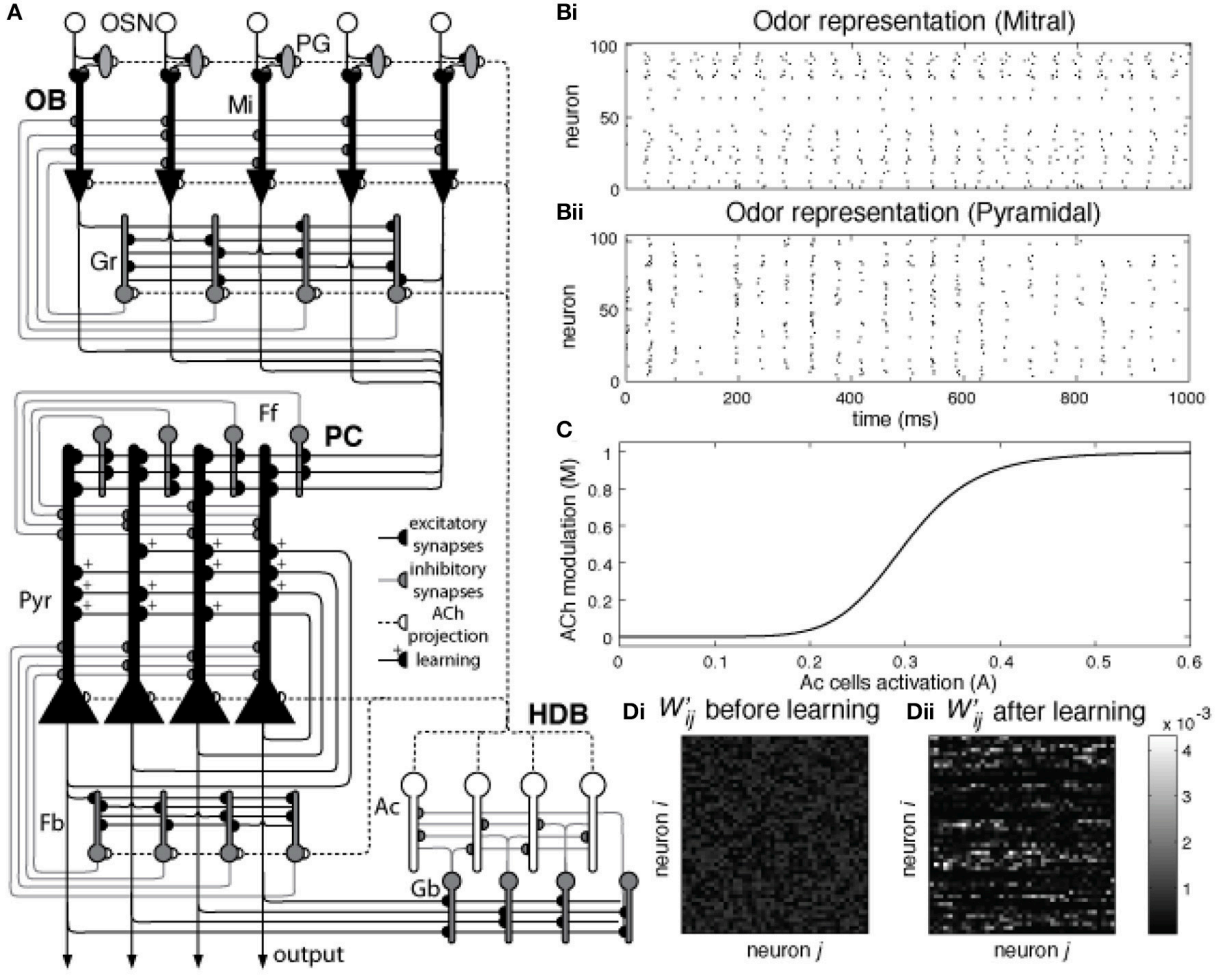
**(A)** Network architecture: Olfactory Bulb, Piriform Cortex, and HDB. Olfactory Sensory Neurons (OSN) connect to one specific glomerulus where they connect with Mitral (Mi) and Periglomerular (PG) cells. Mitral cells are the principal cells within the OB and project their axons to the PC. These neurons are modulated by PG and Granule (Gr) cells (see main text for details). In the piriform cortex, Mi cell axons connect to pyramidal (Pyr) cells and inhibitory interneurons called feed forward (Ff) cells that project their axons to the apical dendrites of Pyr cells, modulating the excitatory input coming from the OB. Pyramidal cells implement an autoassociative network, projecting their axons to other Pyr neurons and to a second group of inhibitory interneurons called Feedback (Fb) cells. In our model, Pyr cells also connect to inhibitory interneurons in the HDB (Gb). These neurons inhibit cholinergic cells (Ac) that project their axons back to the PC and OB, modulating the activity of PG, Mi, Gr, Pyr, and Fb cells. **(B)** Pyramidal cells are randomly connected to Mi cells. The graphs show Mi **(Bi)** and Pyr **(Bii)** activation over a 1 s simulation for a single odorant. Each Pyr cells is connected to 20% of the Mi cells in the OB. Note that mitral cell activities are arranged in neighborhoods for the sake of ease of presentation only. **(C)** Cholinergic modulation in the model depends on Ac cell activation. The graph shows the changes in ACh modulation (M) for different levels of Ac cell activation (A). In our model, average Ac cell activation **(A)** varies between 0 and 0.6 and is described in Equation (2). This activation sets the level of cholinergic modulation in the different neuronal groups and defines how cellular effects in these groups, such as firing threshold, spontaneous activity, suppression of synaptic transmission, etc. are modulated. The relationship between Ac activation and ACh modulation in our model is described in Equation (9). **(D)** Normalized synaptic weights in Pyr autoassociative connections before and after learning. The graphs show the normalized weight matrix $W_{ij}^{\prime }$ for the Pyr-Pyr connections before **(Di)** and after **(Dii)** a set of 9 training sessions. In order to help visualization, only the active synapses are shown. The sum of $W_{ij}^{\prime }$ in both **(Di)** and **(Dii)** is equal to 1, the difference here is that in **(Di)** the weights are spread over most of the active synapses in the network. Over the course of 9 training session **(Dii)**, these weights gradually concentrate in the connections between neurons that are part of the learned odor pattern.

The model implemented here is an extension of our previous work (de Almeida et al., 2013; Devore et al., 2014) in which we explored the relative contributions of ACh modulation in OB and PC. Here, in addition to OB and PC, we implemented a third network representing the HDB (Figure 1A).

The OB network incorporates four neuronal groups: olfactory sensory neurons (OSN), mitral cells (Mi), periglomerular cells (PG) and granule cells (Gr), as described in detail in de Almeida et al. (2013) and shown in Figure 1. Each group is composed of 100 neurons. The PC network is comprised of pyramidal cells (Py), as well as feedforward (Ff) and feedback (Fb) interneurons (Stokes and Isaacson, 2010), each group in this network also consists of 100 neurons. Because the connectivity between OB and PC is still poorly understood, we use random connections between Mi and Pr cells, with probabilities adjusted to match available experimental data (Haberly, 2001; Illig and Haberly, 2003; Davison and Ehlers, 2011; de Almeida et al., 2013; Devore et al., 2014; Nagayama et al., 2014), as shown in Figure 1B. All parameter choices have been explained in detail before and are given in Table 1.

**Table 1:** **List of model parameters**.

General parameters (all neurons)	*v^*hyper*^* = −10 mV; *t^*refrac*^* = 2 ms^*^
Olfactory sensory neuron (OSN)	τ = 5 ms; β = 1; θ^min^ = 0 mV; θ^max^ = 15 mV
Mitral (Mi, apical compartment)^†^	τ = 5 ms
Mitral (Mi, soma compartment)^†^	τ = 5 ms; β = 2; θ^min^ = −1 mV; θ^max^ = 7 mV|3 mV^†^
Periglomerular (PG)	τ = 2 ms; β = 1; θ^min^ = 0 mV; θ^max^ = 10 mV|5 mV^†^
Granule (Gr)	τ = 5 ms; β = 2; θ^min^ = −0.3 mV; θ^max^ = 9.5 mV|7 mV^†^
Feedforward (Ff)	τ = 5 ms; β = 1; θ^min^ = −0.3 mV; θ^max^ = 15 mV
Pyramidal (Pyr)	τ = 10 ms; β = 2; θ^min^ = 0 mV; θ^max^ = 15 mV
Feedback (Fb)	τ = 5 ms; β = 2; θ^min^ = −0.03 mV|−0.13 mV^†^; θ^max^ = 15 mV
Gabaergic (Gb)	τ = 5 ms; β = 3; θ^min^ = 0 mV; θ^max^ = 15 mV
Cholinergic (Ac)	τ = 10 ms; β = 1; θ^min^ = −0.5 mV; θ^max^ = 15 mV
OSN to PG	*g^max^* = 0.166; *E_*N*_* = +70 mV; τ_1_ = 1 ms; τ_2_ = 2 ms
OSN to Mi (apical)	*g^max^* = 0.35; *E_*N*_* = +70 mV; τ_1_ = 1 ms; τ_2_ = 2 ms
PG to Mi (apical)	*g^max^* = 0.38; *E_*N*_* = −10 mV; τ_1_ = 4 ms; τ_2_ = 8 ms
Mi (soma) to Gr	*g^max^* = 0.09; *E_*N*_* = +70 mV; τ_1_ = 1 ms; τ_2_ = 2 ms
Gr to Mi (soma)	*g^max^* = 0.18; *E_*N*_* = −10 mV; τ_1_ = 4 ms; τ_2_ = 8 ms
Mi (soma) to Ff	*g^max^* = 0.2; *E_*N*_* = −10 mV; τ_1_ = 4 ms; τ_2_ = 8 ms
Mi (soma) to Pyr	*g^max^* = 0.76; *E_*N*_* = +70 mV; τ_1_ = 1 ms; τ_2_ = 2 ms
Ff to Pyr	*g^max^* = 0.055; *E_*N*_* = −10 mV; τ_1_ = 4 ms; τ_2_ = 8 ms
Pyr to Fb	*g^max^* = 0.25; *E_*N*_* = +70 mV; τ_1_ = 1 ms; τ_2_ = 2 ms
Fb to Pyr	*g^max^* = 0.55|0.28^†^; *E_*N*_* = −10 mV; τ_1_ = 4 ms; τ_2_ = 8 ms
Pyr to Pyr (association fibers)	*g^max^* = 650|300^†^; *E_*N*_* = +70 mV; τ_1_ = 1 ms; τ_2_ = 2 ms
Pyr to Gb	*g^max^* = 0.085; *E_*N*_* = +70 mV; τ_1_ = 1 ms; τ_2_ = 2 ms
Gb to Ac	*g^max^* = 0.12; *E_*N*_* = −10 mV; τ_1_ = 4 ms; τ_2_ = 8 ms
Pyr adaptation	A^ahc^ = 10|0^*^; *E_*N*_* = −15 mV; τ^ahc^ = 100 ms

* *Spiking neurons*;

† *different values are without|with cholinergic modulation, respectively; The two Mi compartments are electrically coupled and the output computed in the apical compartment is directly applied to the soma compartment*.

The HDB network model is composed of GABAergic (Gb) and cholinergic (Ac) neurons (Brashear et al., 1986). Both types of neurons are known to project to the OB and PC; however the present simulations are only concerned with cholinergic projections to the OB and PC. Cholinergic neurons from the HDB project their afferents to OB and PC and are the main source of ACh in these structures (Záborszky et al., 1986); electrical stimulation of the HDB causes modulation in the PC and OB (Kunze et al., 1991, 1992a,b; Linster et al., 1999; Ma and Luo, 2012) while neural activity in the HDB can be modulated by electrical stimulation in olfactory areas (Linster and Hasselmo, 2000), indicating the existence of a feedback loop between HDB and olfactory areas. Here we assume the interplay between olfactory system and HDB to be similar to the interaction of hippocampus and medium septum proposed in (Hasselmo and Wyble, 1997), where the activity of the medium septum defines the level of ACh modulation in the hippocampus, and the output level of principal cells in the CA1 determine the activation of the medium septum. In our simulations, ACh neurons have strong spontaneous activity, so that the basal levels of ACh in OB and PC are high. The ACh neurons receive inhibitory inputs from local gabaergic interneurons (Gb); these interneurons depend on the activation of Pyr cells to fire and effectively inhibit ACh cells. Therefore, ACh activity is strong if cortical activity is low and gradually decreases as the PC output increases (see results for details).

### Implementation

Our model is composed of single compartment integrate-and-fire neurons, with the exception of Mi cells which are modeled as two compartments. The equations defining these neurons are adapted from previous models (Linster et al., 2007, 2009; de Almeida et al., 2013; Devore et al., 2014; Mandairon et al., 2014). Changes in membrane voltage v(t) over time in each compartment are described by Equation (1):

(1) $$\begin{array}{l} \tau \frac{dv\left(t\right)}{dt}+v\left(t\right)=V^{ext}\left(t\right) \end{array}$$

where τ is the membrane time constant and *V*^*ext*^(*t*) is the voltage change resulting from external input over time.

Each one of the voltage changes due to external inputs *V*^*ext*^ is a result of the synaptic strength of the connection from neuron *j* to neuron *i* (*w*_*ij*_) and the conductance in cell i at time t (*g*_*i*_(*t*)). *E*_*N, ij*_ is the Nernst potential of the synaptic current and *v*_*i*_(*t*) is the membrane potential of the postsynaptic neuron *i*, as described in Equation (2):

(2) $$\begin{array}{l} {V_{i}}^{ext}\left(t\right)=w_{ij}g_{i}\left(t\right)\left[E_{Nij}-v_{i}\left(t\right)\right] \end{array}$$

The communication between neurons can be either continuous or discrete, depending on the type of presynaptic neuron. Our network is composed of non-spiking (firing-rate) neurons (OSN and PG cells) and discrete spiking neurons (all the others). Non-spiking neurons represent large populations of neurons assumed to have similar activity over time. The output *F(v)* of a given neuron *i* is a function of its membrane potential *v* and the minimal threshold and saturation threshold of the output function, θ^*min*^ and θ^*max*^. Where *F*_*i*_(*v*) = 0 if *v* ≤ θ^*min*^ and *F*_*i*_(*v*) = 1 if *v* ≥ θ^*max*^. For values between zero and 1 *F*_*i*_(*v*) is given by Equation (3):

(3) $$\begin{array}{l} F_{i}\left(v\right)=\left(\frac{v-\theta^{min}}{\theta^{max}-\theta^{min}}\right)\beta \end{array}$$

where β defines the non-linearity of *F*_*i*_(*v*). For non-spiking neurons, *F*_*i*_(*v*) represents the continuous activation rate of the cell, while in spiking-neurons, *F*_*i*_(*v*) defines their instantaneous firing probability. The time course of the conductance change is calculated as:

(4) $$\begin{array}{l} gi_{\left(t\right)}=g^{max}\left(e^{-\frac{t}{\tau_{1}}}-e^{-\frac{t}{\tau_{2}}}\right) \end{array}$$

where *g*^*max*^ is a constant with no unit representing the maximum conductance of a given channel, while τ_1_ and τ_2_ are the rising and falling times of this conductance. After firing, the spike of each spiking-neuron is reset to a hyperpolarization potential *v*^*hyper*^ and remains inactive for a refractory period *t*^*refrac*^.

Spike adaptation was implemented in Pyr cells as a change in voltage ${\text{v}}_{i}^{ahc}$ (t) due to a hyperpolarizing current that increases the firing threshold for recently active neurons when cholinergic modulation is low:

(5) $$\begin{array}{l} \tau^{ahc}\frac{d{v_{i}}^{ahc}}{dt}+{v_{i}}^{ahc}=A^{ahc}X_{i} \end{array}$$

where *X*_*i*_ is equal to 1 in the time-step after neuron *i* spikes and 0 otherwise. Therefore, $v_{i}^{ahc}$ increases with the constant *A*^*ahc*^ and decays with the characteristic time τ^*ahc*^.

The weight changes in Pyr cell associative connection are implemented in our network as a Hebbian learning rule, the synaptic strength *w* will be enhanced if both pre and postsynaptic neurons fire together, as shown in Equation (6):

(6) $$\begin{array}{l} \frac{dw_{ij}}{dt}=M\left(1-w_{ij}\right)\frac{i^{post}\left(t-t_{j}^{fire}\right)b^{glu}\left(t-t_{j}^{fire}-t^{delay}\right)}{\tau^{pp}} \end{array}$$

where *M* is the level of ACh modulation, defined in Equation (8). *w*_*ij*_ is the synaptic weight, *t*^*delay*^ is the time it takes for the action potential to travel from the soma to the recurrent connections, *i*_*post*_ is the postsynaptic depolarization as defined in (1) and *b*^*glu*^ is the time course of glutamate binding on NMDA receptors. Changes in synaptic enforcement have been long attributed to the co-activation of these mechanisms (Bliss and Collingridge, 1993) and *t*^*pp*^ is the time constant of synaptic change. *b*^*glu*^ is defined by:

(7) $$\begin{array}{l} b^{glu}\left(t\right)=\exp\left(-\frac{t}{\tau^{NMDAf}}\right)\exp\left(-\frac{t}{\tau^{NMDr}}\right) \end{array}$$

where τ^*NMDAf*^ and τ^*NMDAr*^ characterize the receptors' kinetics.

The weights of associative synapses are initially set to a random value between 0 and 0.04, which is ~10% of the maximum weight.

### Cholinergic modulation

Cholinergic modulation impacts a wide range of cellular and synaptic properties in cells of the OB and PC. Levels of ACh concentration are assumed to be a function of the average Ac activity in the HDB, as shown in Figure 1C, and uniformly distributed across the whole olfactory system. Therefore, Ac activity sets the level of ACh modulation *M* in the network, as described by Equation (8):

(8) $$\begin{array}{l} M=\frac{1}{1+{\left(\frac{Y_{1/2}}{A\left(t\right)}\right)}^{\beta ACh}} \end{array}$$

here, *Y*_1/2_ is the activation at which half-maximal modulation would be achieved, *A(t)* is the average activation of Ac cells at time *t* and β^*ACh*^ defines the non-linear effects of Ac activation observed in dose-response curves (Liang et al., 2005). Cellular cholinergic effects, such as changes of synaptic transmission, spontaneous activity, cellular adaptation, etc. vary linearly with *M*. These effects are implemented in the model through changes in different neuron parameters. The complete list of parameters used in is found in Table 1. Finally, the changes in the activation *A*(*t*) are described in Equation (9):

(9) $$\begin{array}{l} \tau^{ACh}\frac{dA\left(t\right)}{dt}+A\left(t\right)=\overset{n}{\underset{i=1}{\sum }}S_{i}\left(t\right) \end{array}$$

where τ^*ACh*^ is the time constant of cholingeric levels in the system *n* is the number of Ac cells in the network and *S*_*i*_(*t*) is the state of neuron *i* at time *t*, this state can be 1 (firing) or 0 (otherwise). As a consequence. levels of ACh present in the OB and PC vary slowly as a function of HDB cholinergic neural activity.

The effects of ACh on OB and PC cells can be found in more detail in previous work (de Almeida et al., 2013), and see Table 1 for a complete list of parameters.

### Analysis

#### Sparseness

The selectivity of odorant activation is measured using a measure for sparseness in the neuronal response (Rolls and Tovee, 1995; Poo and Isaacson, 2009), as defined by Equation (11):

(10) $$\begin{array}{l} S=\;1-\frac{1-\left({\left(\sum_{i=1}^{N}\frac{R_{i}}{N}\right)}^{2}/\sum_{i=1}^{N}\frac{R_{i}^{2}}{N}\right)}{1-1/N} \end{array}$$

where *R*_*i*_ is the average firing rate of cell *i* when exposed to a given odor pattern, and *N* is the total number of cells. A response is highly sparse (*S* = 1) when a single cell is active, while it has minimal sparseness (*S* = 0) when all cells have the same activity.

#### Coherence

The measure of synchronization adopted here was proposed in de Almeida et al. (2013) and defines the level of coherence between two neurons *i* and *j* as the cross-correlation between this pair of neurons when compared to the cross-correlation that would be observed in random spiking neurons *i'* and *j'*, with the same firing rates of neurons *i* and *j*, as described by Equation (11):

(11) $$\begin{array}{l} c_{ij}={\left[1-\frac{\sum_{l=1}^{K}X_{i}^{\prime }\left(l\right)X_{j}^{\prime }\left(l\right)}{\sum_{l=1}^{K}X_{i}\left(l\right)X_{j}\left(l\right)}\right]}_{+} \end{array}$$

where [*x*]_+_ = max(0,*x*) indicates linear rectification. *X*_*i*_(*l*) = 0 or 1 indicates the event of a spike in neuron *i* in the time bin [(*l-1)*t, *l*t] where *l* = 1, 2, 3, …, *K*, t = *T*/*K* = *2 ms* and T is a long time interval. For the entire neuron population, the coherence *c* is defined by the average of *c*_*ij*_ over many pairs of neurons in the network, so that *c* is 0 when the network shows no synchronization and progressively higher as neurons synchronize.

#### Distance

The similarity between two odor representations is measured as the distance between the neuronal activities evoked by these odorants. This distance D_*O*1, *O*2_ is calculated as one minus the normalized dot product between activity vectors (*O*_1_ and *O*_2_) with the average activity resulting from odor presentation for 1 s, as shown in Equation (12):

(12) $$\begin{array}{l} D_{O_{1}O_{2}}=1-\frac{\sum_{i=1}^{N}O_{1i}O_{2i}}{\left||O_{1}||||O_{2}|\right|} \end{array}$$

where *O*_1*i*_,*O*_2*i*_ are the elements of the activity vectors *O*_1_ and *O*_2_, respectively, and ||*O*_1_||, ||*O*_2_|| are the norms of vectors *O*_1_ and *O*_2_. Thus, the value of *D* is close to zero when the activity vectors *O*_1_ and *O*_2_ are similar and increases as this similarity is reduced.

#### Odor stimulation

Different odors can be defined by a distribution of affinities across the OSNs. In our model, these affinities are represented by the activation of OSNs elicited by a given odorant, so that, the higher the activation, the higher the odor affinity, similar to de Almeida et al. (2013). For the tests presented here, we generate 100 different affinity values that are randomly permutated to represent each odorant. This method insures that all odors impose the same average input to the OSN layer. The numerical values of the affinities are calculated from the normal probability density function N(x,μ,σ) for, *x* = 1, 2, …, 100 (the total number of OSNs in our network), with μ = 50 and σ = 10 and directly define OSN outputs. As a result, an odor stimulation is characterized by a distribution of OSN outputs ranging between 0 and 1. For the simulations reported in the results section, affinity values for each odorant are randomly distributed across the OSN population. In many of these results, the similarity (distance, Equation 12) between two odorants is an important factor. In order to better control the level of similarity between odor, we initially created and stored a pool of 2000 randomly generated odorants. Each one of these odors were then simulated and their Pyr activation patterns were stored. We then perform pairwise comparisons of these activation patterns using Equation (12).

### Implementation

All simulations were implemented using the *Matlab* programming language, with Euler integration method for the differential equations with a time step of 0.5 ms. The source code of these models can be downloaded from the modelDB website (Hines et al., 2004) at the link senselab.med.yale.edu/ModelDb. The correlations were measured using Spearman's rank correlation and the *p*-values of these correlations were calculated using large sample approximations.

### Electrophysiology

**Figure 2:**
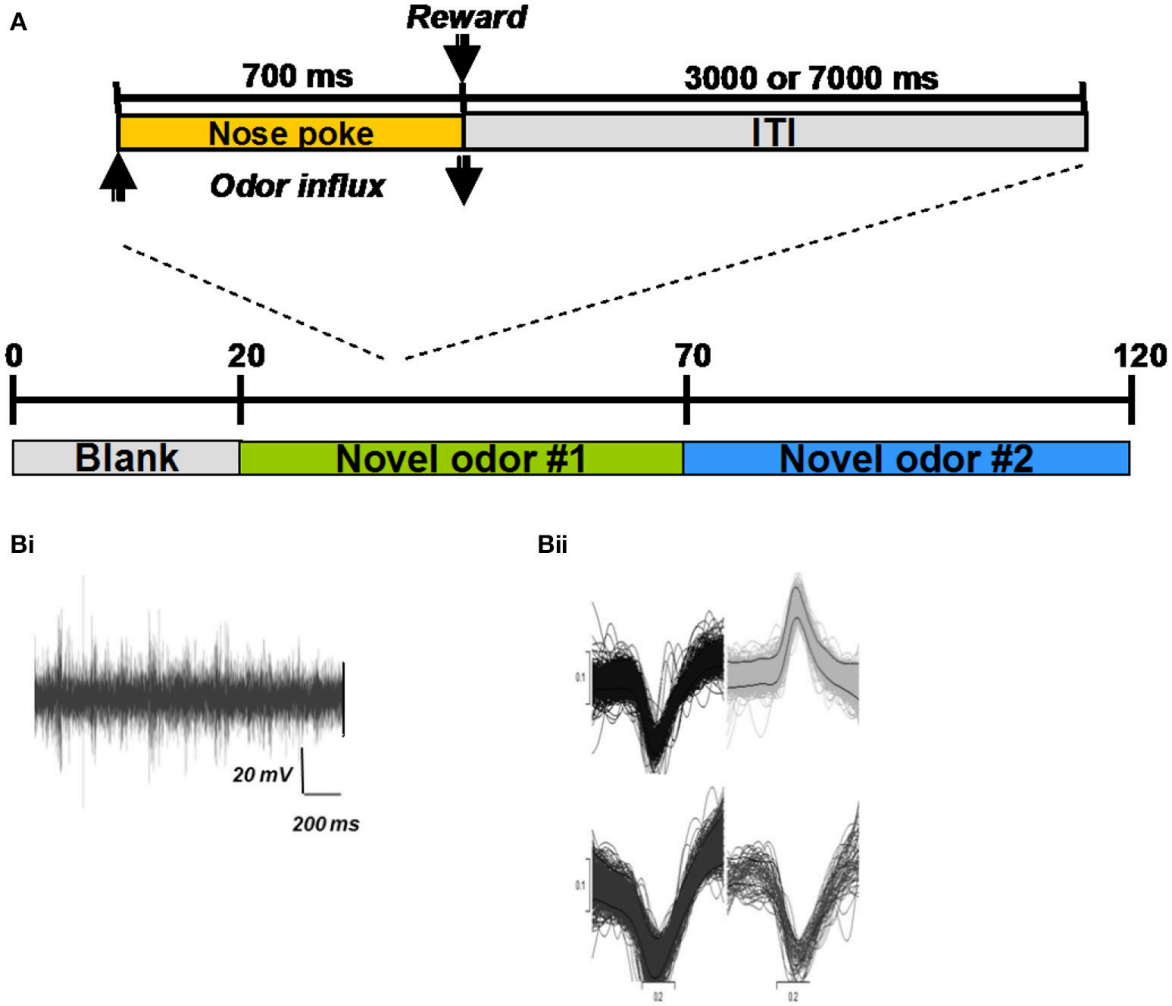
**Electrophysiological experiments and example recordings. (A)** All behavioral training and testing took place in an operant chamber (Coulbourn Instruments, Whitehall, PA). Rats were trained to poke their nose into an odor port located centrally in the front wall of the chamber and hold for at least 700 ms (Figure 1A; inset). An infrared photobeam detected entry into the nose port and triggered delivery of odors (see details of odor delivery in Devore et al., 2014). If the rat withdrew from the odor port after less than 700 ms, no reward was given and the trial was counted as incorrect. If the rat withdrew his nose after more than 700 ms, a sugar pellet reward was dropped into the glass dish and the 3000 ms ITI started. All hardware events were controlled using custom-written Labview (National Instruments, Austin, TX) routines. For each session, 20 blank trials were followed by 50 trials with a novel odor (#1) and 50 trials with a second novel odor (#2) **(Bi)** Example recording trace from a single electrode in the HDB. **(Bii)** Examples of extracted spike shapes from a recording electrode.

Two adult male Long Evans rat (350 gm) were used in this study. Rats were allowed unlimited access to water but were food deprived to 85–90% of their *ad libitum* body weight. To test how neural responses in the HDB change as an odor becomes increasingly familiar, rats were trained to perform a simple nose poke task in a Colburn operant conditioning box connected to an olfactometer (Figure 2A; for details on olfactometer design see Devore et al., 2014). Briefly, rats were trained to poke an odor port and sample the odorant for a minimum of 700 ms for a food reward (sugar pellet), and to respect a 3 s intertial interval. If the rat failed to reach the minimum 700 ms nose-poke duration, the lights in the chamber would turn off and the ITI would increase to 7 s (Figure 2A). This trial was considered a failure. The rat was considered sufficiently trained when it achieved 90% correct trials. A correct trial simply meant that the rat kept its nose in the port for the required time. Rats were not trained to discriminate odors in this task, our goal was merely to have rats become familiar with an odor and form an association between the odor and reward. During behavioral testing, rats first poked in response to a blank stimulus to gather baseline data, followed by 50 trials with a first odor and 50 trials with a second odor to measure how activity changed as an odor became familiar. Using a blank followed by two novel odorants allowed us to record baseline activity, to monitor how familiarity changed neural activity and to test, using a second odor, if any observed changes were just due to time or were indeed related to odor familiarity.

After training, rats were implanted with one monopolar 50 um stainless steel electrode and two microdrives in the mitral cell layer of the OB, anterior PC, and HDB, respectively under isoflurane anesthesia. Each of the microdrives consisted of a single 25 um stainless steel tetrode that could be raised and lowered after surgery. Following a week of recovery, rats were retrained on the behavioral paradigm back to pre-surgical levels. Following retraining, on each recording day, rats were first plugged into the recording rig and allowed to freely roam around a 1 × 1 m open-field box for at least 10 min. Rats were then placed in the operant conditioning box and run on a novel odor set each day. Each session consisted of 20 no-odor trials, 50 trials with odor 1 and 50 trials with odor 2 (see Table 2 for odorsets).

**Table 2:** **Odors used in electrophysiology experiments**.

**Recording day**	**Odor block 1**	**Odor block 2**
1	Limonene	1,8 cineole
2	2,2-pheyl alcohol	(+) carvone
3	3 propanal ethyl propionate	4 tans-2-nonenol methyl valerate
4	Hexyl butyrate	Methyl valerate
5	n-amyl acetate	Methyl butyrate

Neural activity was recorded through an HS-27 head stage connected to a Neuralynx ERP-27 Panel with a Lynx-8 Amplifier (Neuralynx, Inc., Tucson, AZ, USA). The raw signals were amplified and filtered to isolate spiking activity (5000 X, 600–6000 Hz), digitized at a sampling rate of 20 kHz using a CED Power1401 and Spike2 software (Cambridge Electronic Design, Cambridge, United Kingdom) and stored onto a computer disk for offline analysis (Figure 2B). At the end of each session, electrodes were advanced by rotating the drive legs either 1/8 or 1/4 turn; a complete revolution lowered the electrodes by 320 μm. Single units were extracted from raw waveforms using the Spike2 spike-sorting package, which uses principal components analysis as well as waveshape features. Electrode locations were verified histologically at the end of the experiment. All animal experiments were conducted under a protocol approved by the Cornell Institutional Animal Care and Use Committee.

For the purposes of this report, neural activity recorded from HDB electrodes during odor behavioral sessions was analyzed (Devore et al., 2016). Only well isolated units for which the amplitude individual action potentials were at least two standard deviations above the average activity were included in the analysis presented here. We first calculated the spontaneous activity of each extracted neuron from the first 20 trials during which rats were sampling the odorless port. For each neuron, we then tested (1) wether activity was significantly modulated by odor stimulation during the odor sampling periods and (2) how overall activity changed as rats became more familiar with the odors presented during a session. A neuron was considered “responsive” to odor stimulation if activity during the 2000 ms after the start of odor sampling was significantly lower or higher than during the 2000 ms preceding the start of odor sampling (*t*-test). For each odor in a session, we compared the activity of each neuron during the first 50 s of odor sampling trials to that during the last 50 s of odor sampling trials to test if the neuron's activities changed significantly over the course of the rat sampling that odor. The relationship between spontaneous firing rate and a significant decrease or increase between the first and last 50 s of odor trials was tested using Pearson's correlation.

## Results

### Bi-directional regulation of odor learning, contrast enhancement and levels of ACh

Acetylcholine has been shown to improve LTP on recurrent connections between Pyr cells, effectively promoting learning (Williams and Constanti, 1988; Hasselmo and Barkai, 1995). In our previous models, odor learning resulted in a gradual increase in activity of odor responsive Pyr cells. We first test how the learning process in our new model affects ACh modulation. We then investigate how this modulation impacts different cell groups and odor representations over a sequence of training sessions.

**Figure 3:**
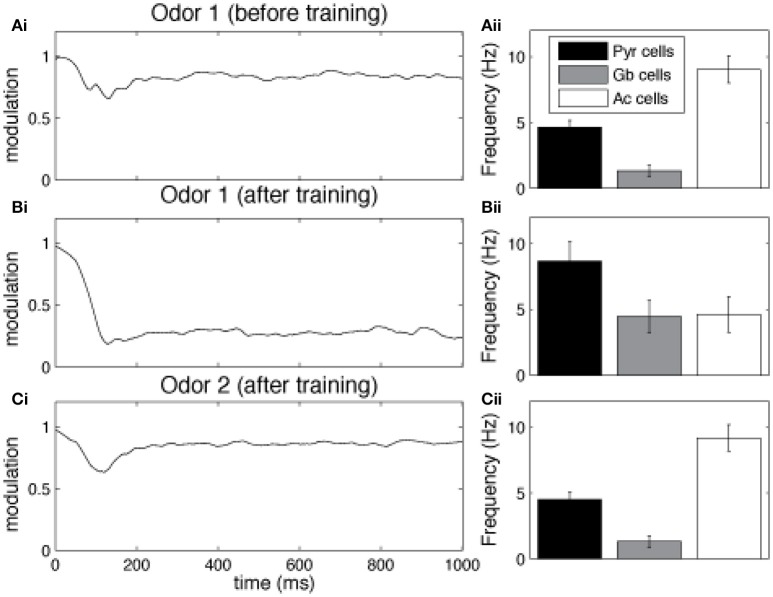
**Cortical learning dynamically changes the level of ACh modulation in the model**. The graphs show the average level of ACh modulation in the system over 100 different 1 s simulations. In all tests, the level of modulation M depends on Ac cell activation (see Figure 1) and is initially set to max (1). Ac cells are indirectly inhibited by Pyr activation through Gb neurons in the HDB. Therefore, high Pyr activation leads to low Ac firing (see methods for details). **(A)** Cholinergic modulation is kept high for unknown odor patterns. The graph shows that for unknown (not yet encoded) odors (odor 1), Pyr activation is low, since the autoassociative connections between active neurons are not strong. Low Pyr firing results in low Gb cell firing **(Aii)** and high Ac activation and high levels of ACh modulation **(Ai)**. This high modulation, however, sets the system into “*learning mode*,” by gradually promoting LTP between Pyr autoassociative connections. **(B)** Cholinergic modulation is drastically reduced for learned odor patterns. The graph shows the levels ACh modulation after nine training sessions using the same odor pattern **(Bi)**. The sequence of training sessions helps to increase the synaptic weights of associative connections between Pyr neurons recruited by the odor pattern (odor 1). When odor 1 is presented, Pyr cells are activated, by both external inputs from OB and enforced recurrent connections with other Pyr cells **(Bii)**. This stronger firing rate indirectly inhibits Ac neurons through Gb cells and reduces the overall ACh modulation in the model, The lower ACh modulation increases the efficiency of autoassociative connections and switches the system to a “*recalling mode*.” **(C)** Presenting a new unknown odor dynamically switches the system back to “*learning mode*.” In **(Ci)** the graph shows the level of ACh when the network is stimulated with a novel odor (Odor 2) after learning Odor 1. In this example, Odors 1 and 2 are kept very different, so that their activity patterns don't share Pyr cells (see examples in Figure 1B). The associative connections between the activated Pyr cells for this new activity patterns are not strengthened, resulting in low overall Pyr and Gb firing, unable to inhibit Ac activation **(Cii)**. This keeps ACh modulation high and moves the system back to “*learning mode*.”

Cortical learning dynamically changes cholinergic modulation levels in the network (Figure 3). The model is presented with a previously unknown odor pattern (odor 1) for a sequence of nine 1 s training sessions, subsequently, we present either the trained odor or a second, unknown odorant to the network (odor 2). In response to a novel odor input, the level of modulation is initially high (1.0), since the Ac cells in our HDB exhibit strong spontaneous activity (Figure 3Ai). After ~80 ms, the increased activation of Pyr cells (due to synaptic plasticity) partially inhibits Ac cell firing and overall ACh modulation is slightly reduced. At the same time, the high ACh modulation is setting the Pyr network into “learning mode,” hence gradually promoting LTP between Pyr autoassociative connections. The graph in Aii shows the average activity of pyramidal (Pyr), gabaergic (Gb) and cholinergic (Ac) cells during the first second of the nine second training session. After an odor has been learned, recurrent synapses between odor responsive Pyr cells are strengthened and as a result the overall firing rate of odor responsive Pyr cells is dramatically increased (Figures 3Bi,Bii), indirectly inhibiting Ac neurons in the HDB and reducing the overall ACh modulation in the system. The reduced ACh modulation decreases synaptic plasticity and restores synaptic transmission of the Pyr autoassociative connections to 100%, effectively switching the Pyr network to a “recall” mode. When a novel, untrained odor is presented (Figures 3Ci,Cii, Odor 2 after training of odor1), the previously learned pattern is not reactivated, Pyr firing is low, ACh inputs are high, effectively switching the cortical network to “learning” mode.

**Figure 4:**
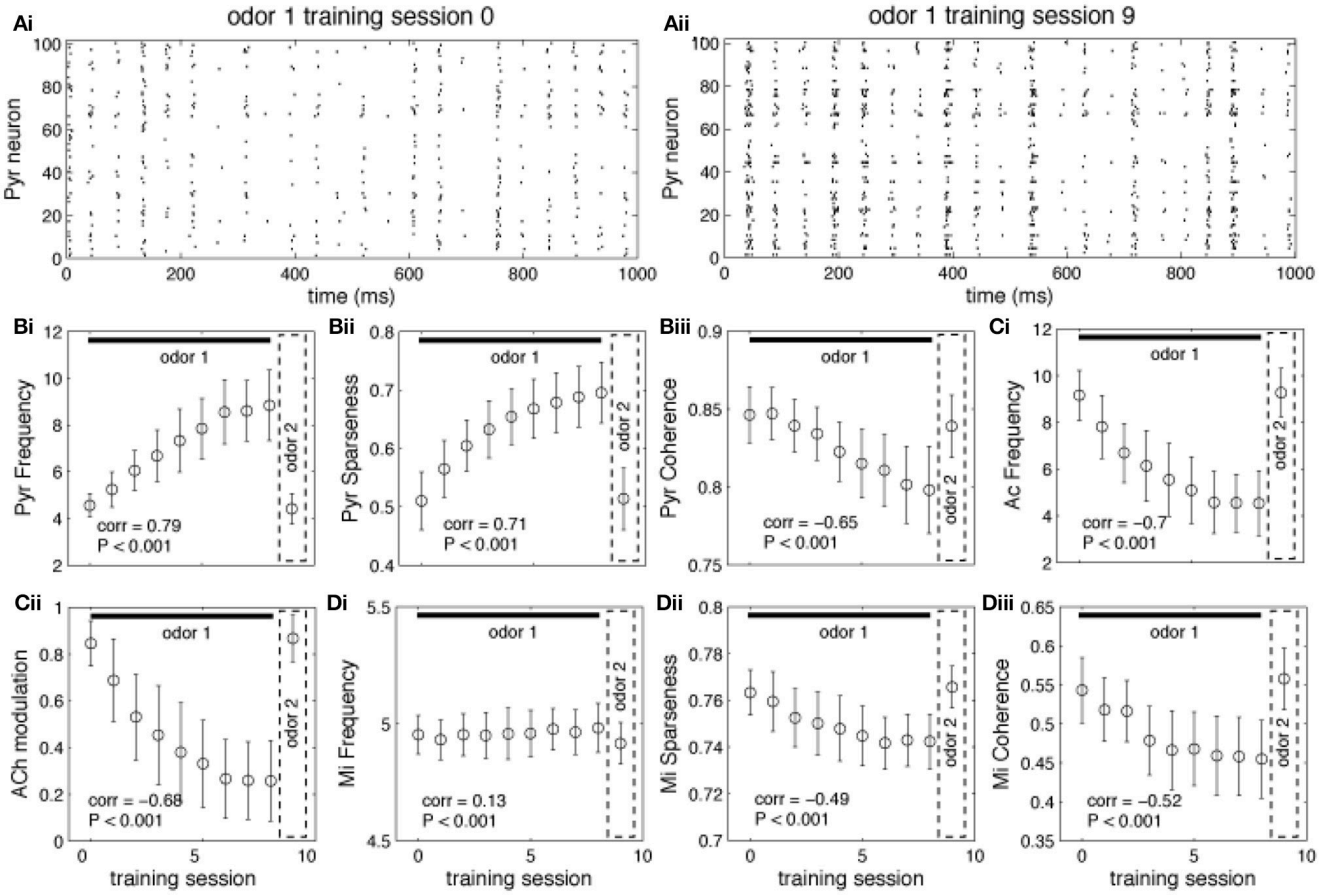
**Regulation of learning. (A)** Pyr network activation before and after learning. The graphs show raster plots of the Pyr network before **(Ai)** and after **(Aii)** learning one odor pattern. The differences in firing rate between **(Ai)** and **(Aii)** results from strengthening the synaptic weights between neurons responding to the odor. **(B,C)** Effect of learning on PC and HDB neural activity. The graphs show how the average frequency of Pyr and Ac cells varies over a sequence of training sessions. The last data point in each graph shows the average response to a novel, untrained odor. The overall firing rate of Pyr cells increases as the synaptic weights between Pyr cells are incremented over the sequence of training sessions **(Bi)**. At the same time, the indirect inhibition of Ac cells by Pyr activity is also increased, reducing Ac activation and ACh levels **(Ci,Cii)**. Activity levels return to baseline when a new odor pattern (odor 2) is presented. Learning improves Pyr sparseness **(Bii)** by increasing the autoassociative connection weights of neurons that participate in the odor representationPyramidal coherence **(Biii)**, on the other hand, slightly decreases as a result of the decrease in Mi coherence when ACh modulation decreases (see **Diii**). **(D)** Effect of cortical learning on OB activity. The graphs show how Mi frequency **(Di)** sparseness **(Dii)** and synchronization **(Diii)** are affected by the change in ACh levels resulting from learning. Mi cell firing rates are only weakly affected by learning in our model; however, ACh modulation changes Mi sparseness and synchronization **(Dii,Diii)**. Correlation values on each plot show the correlation between each measure and the learning sessions (Pearson's r).

Graphs in Figure 4 show how neural activity in the network changes as an odor is learned and the network gradually moves from “learning” to “recall.” Each point represents the average of 100 simulations with randomly chosen odors, while the bars measure the standard error. As an odor is learned, Pyr cells increase their firing in response to the odor because association fibers are strengthened and Pyr cells receive increased inputs from each other (compare Figures 4Ai,Aii and see also Figure 3Bi). At the same time, the learning process leads to sparser representations of odor 1 in cortical cells (Figure 4Bii) as described previously (de Almeida et al., 2013). Overall, the odor representation in the cortical network is stronger and more specific as a result of synaptic plasticity. As the odor is learned, activity in cholinergic cells (Figure 4Ci) decreases, and with it the amount of ACh modulation in the OB and PC (Figure 4Cii). Odor responses of OB mitral cells are affected by the decrease in cholinergic tone, as evidenced by small changes in firing rates (Figure 4Di), and larger changes in sparseness (Figure 4Dii) and synchrony (Figure 4Diii), as described previously. Cortical learning strongly affects bulbar odor representations via regulation of cholinergic inputs: cholinergic input ensures sparse and synchronous bulbar inputs to PC during learning but not recall.

### Cortical attractors determine switch between learning and recall

Learning in associative networks creates representations in cortical neurons that can be more or less specific to the learned pattern, depending on learning parameters. In most cases, a certain degree of variation from the original pattern will allow for reconstruction, or completion of the original pattern, making the memories robust to noisy or distorted inputs. The degree of distortion enabling reconstruction depends on the size of the basin of attraction that has been formed. We here test how dissimilar a “novel” odor has to be as compared to a previously learned odor to allow the cortical network to enter the “learning” mode rather than staying in “recall” mode. We first train the network with a novel, randomly chosen, odorant (odor 1) for nine sessions, then we present a different, randomly chosen, odorant (odor 2) in the 10th session. We measure whether the network will be in recall or learning mode as a function of the distance (Equation 12) between the learned and unlearned odors at the input to the olfactory bulb.

**Figure 5:**
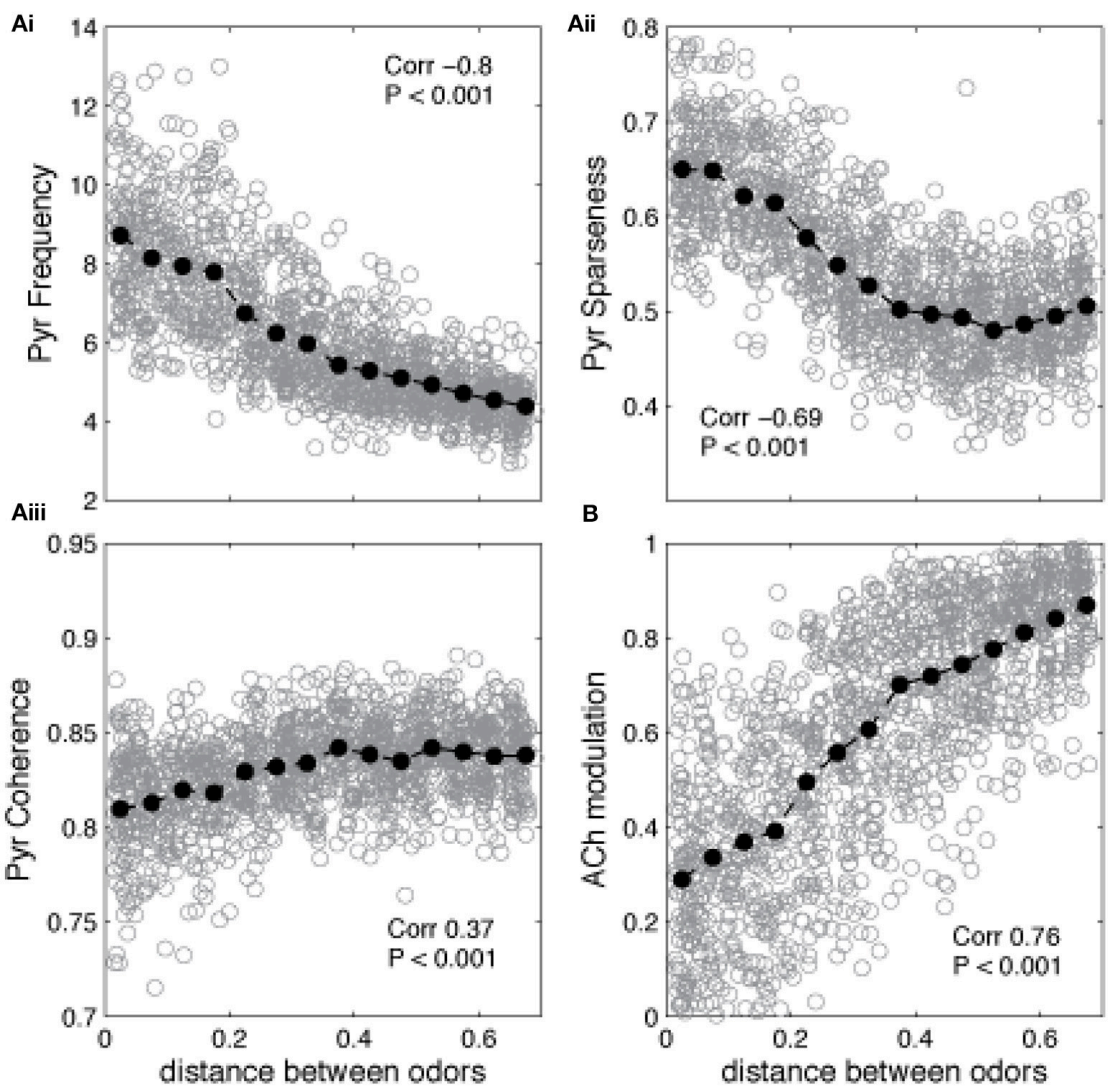
**Similarity between learned and recalled odor patterns impacts the regulation of encoding and recall**. The graphs show how Pyr average frequency, sparseness and coherence—as well as cholinergic modulation—are affected by the level of similarity between a previously trained odor and a novel odor. The gray circles show individual data points, while the black filled circles show the average values. The correlation values indicate the correlation between the plotted measure and the distance between the two odor representations (Pearson's R). **(Ai)** Pyramidal cell firing rates in response to a novel odor decrease as the distance between encoded and novel odor increases. Small distances indicate that the overlap between the two odors is high. As the distance increases and the overlap is reduced, the network cannot rely on the learned connections to recall the learned odor and the PC responses to the novel odor decreases. A similar trend is observed for the sparseness of Pyr firing, which also depends strongly on learned association fibers **(Aii)**. Finally, coherence is only slightly reduced by the overlap between trained and evoked odors **(Aiii)**. Changes on Pyr coherence depend on Mi coherence, which are positively correlated with the levels of ACh **(B)**.

The state of the network in response to presentation of a novel odor AFTER learning of a different odor defines whether the network is in learning or recall mode: if ACh modulation is high and Pyr frequency is low, the system is in learning mode (the novel odor will be learned), if ACh modulation is low and Pyr frequency is high, the system is in recall mode (this means that the novel odor is similar to the learned odor and falls into its basin of attraction). The graphs in Figure 5 show the average state of the network during the first presentation of a novel odor after training as a function of the distance between trained and novel odor. As described above, the state of Pyr response and ACh modulation levels define if the network goes into learning or recall mode. For small distances (i.e., similar training and recalling odors) the average Pyr frequency in response to the novel odor is high, since the overlap between the odorants is high (Figure 5Ai). As a consequence, the system is in recall mode. As distance increases, the network cannot rely on the learned connections anymore, firing frequency is reduced, which leads to a reduction on Gb cell activation in our HDB model and an increase of ACh modulation. Similar effects are observed for the level of sparseness. For small distances (high overlap), the attractor created by the learned memory keeps the odor representation sharp, by activating the cells that are part of the trained odor pattern (Figure 5Aii). Cortical sparseness rapidly decreases when the similarity between trained and novel odor is reduced while the level of coherence is only slightly affected (reduced) by the overlap between trained and evoked odors (Figure 5Aiii). The level of ACh modulation varies ~3 × (from ~0.27 to ~0.85), while Pyr activity changes ~2 × (between ~4.2 and ~8.6Hz), suggesting that ACh modulation is more affected by the overlap between odor representations then the average frequency. This higher sensibility to overlap derives from the non-linear relationship between Ac cell activation and ACh modulation, described in Equation (1) (see Figure 1C) and improves the dynamics of the system, allowing for relatively small variations of Pyr frequencies to trigger large changes of ACh modulation. In summary, given the parameters for learning used here, the cortical network induced switch between recall and learning occurs for odors that are more distant than 0.2 using our distance measure.

### Neural activity in the HDB changes as odors become familiar

**Figure 6:**
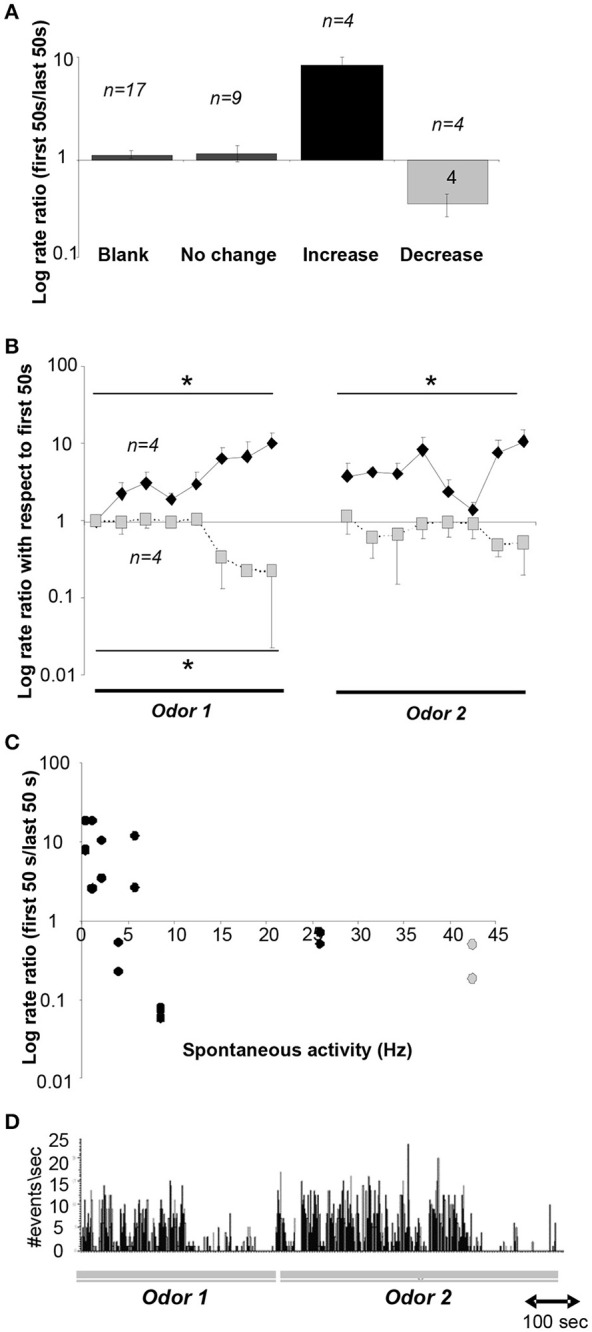
**Neural recordings in the HDB. (A)** Average ratio of neural activity during the first and last 50 s of an odor sampling period. The y-axis is plotted on a log-scale for ease of visualization. Data shown are responses during the 20 presentations of the blank for all neurons, and responses during both odor sampling periods for neurons that showed no change, an increase or decrease. **(B)** Time course of neural activity for neurons showing a significant increase (*n* = 4) or decrease (*n* = 4) over the course of the sampling of odor 1 and odor 2. Because animals we free to sample as they wished, the time course of sampling was divided into 8 equal time intervals for each animal. ^*^indicate significant differences between time points. The y-axis is plotted on a log-scale for ease of visualization. **(C)** Degree of increase/decrease in each neuron as a function of spontaneous activity measured during sampling of the blank odor. Decreases in activity are seen throughout the range of spontaneous activity frequencies observed. The y-axis is plotted in a log scale for ease of visualization. **(D)** Example activity of a neuron during sampling of odors 1 and 2.

Our simulations predict that as a novel odor becomes more familiar to the animal, the activity of cholinergic neurons in the HDB network would decrease from the level of spontaneous activity whereas at the same time, the activity of GABAergic neurons would increase. To test how neurons in the HDB change their firing rates as a rat becomes familiar with an odorant, we recorded from HDB neurons, putatively including cholinergic and GABAergic neurons since cannot be identified in extracellular recordings, during a simple odor-reward association task (see Methods and Figure 2). We analyzed data from 17 neurons in two rats, each run in four sessions with two novel odors. These neurons exhibited spontaneous activities ranging between 0.2 and 42 Hz with an average rate of 14.122 ± 3.4 Hz. These data are in the range of those reported in a previous report in urethane anesthetized (Linster and Hasselmo, 2000): 0.5–16 Hz with an average of 6.7 ± 1.19 Hz) and awake behaving rats (Devore et al., 2016; 11.05 ± 11.02 Hz). 70% of these neurons (*n* = 12) fired significantly differently during odor sampling than before odor sampling; in the majority of cases (*n* = 9) neurons decreased their firing rates during sampling suggesting the possibility of an anticipatory increase before odor port sampling. We then tested if neural activity of HDB neurons changed over the course of familiarization with the odorants by comparing average activity during the first and last 50 s of each odor sampling period. We found that neural activity either significantly decreased (24%), increased (24%) or did not change (53%) between the first and last 50 s of an odor sampling period (Figure 6). Figure 6A summarizes the degree of change in response to odorants over the course of familiarization in response to blank and odors. In each case, the activity during the first 50 s is compared to the activity during the last 50 s. The data are separated into neurons decreasing their activity (*n* = 4), increasing their activity (*n* = 4) or showing no change (*n* = 9). Figure 6B shows the temporal evolution of average firing rates of decreasing and increasing neurons during the 50 trials odor sampling period for each odor in 8 samples of equal duration. All firing rates were first normalized with respect to the first sample of odor A to allow comparisons between neurons. Because rats are self-guided in this behavior, they run through the 50 trials for each odor with unequal time, as a consequence each odor period was divided into eight equal samples rather than taking samples of equal time lengths. Note that on average, the activity returns to the baseline firing rate when the novel odor B is first presented, showing that familiarity with each odorant modulates the neurons' firing rates. A significant correlation between spontaneous firing rates and degree of change was found for the 8 neurons that changed their firing rate over the course of familiarization with an odorant (*r* = −0.476; *p* <0.05; Pearson Correlation; Figure 6C). Figure 6D shows an example for a cell responding with decreased firing over the course of sampling odor 1, increased firing followed by a decrease when odor 2 is presented.

## Discussion

Cholinergic modulation has long been suggested to provide a switch between encoding and recall modes in cortical or hippocampal associative memory (Hasselmo and Bower, 1993). The activation of cholinergic receptors increases pyramidal cell excitability and enhances LTP, allowing cortical networks to undergo a period of high plasticity necessary for memory formation. Theoretical ideas suggesting a switch from focusing on incoming information (encoding) or stored information (recall) are supported by experimental data showing a dependence of learning, but not recall of information on functioning ACh receptors (Hasselmo et al., 1992; Hasselmo and Bower, 1993; Yu and Dayan, 2005; Hasselmo and Sarter, 2011). The olfactory system is served by a separate, mostly exclusive cholinergic nucleus, and hence lends itself well for detailed investigations about the role of ACh in learning and recall (Brashear et al., 1986; Záborszky et al., 1986). Behavioral experiments by DeRosa and colleagues showed that encoding (learning) of odor information, especially in ambiguous situations, is highly dependent on activation of cholinergic receptors (De Rosa and Hasselmo, 2000; De Rosa et al., 2001, 2004). Similarly, Linster and colleagues showed that encoding of olfactory information was impaired when cholinergic inputs to the olfactory system were decreased (Devore et al., 2014). These results are in agreement with data from other systems, such as hippocampus and sensory cortex, where encoding of overlapping or ambiguous information is enhanced by cholinergic modulation (Baxter and Chiba, 1999; De Rosa et al., 2001; Ljubojevic et al., 2014). Experiments from our and other labs have shown that cholinergic modulation in the olfactory bulb regulates the similarity between odor representations, and that this regulation is specifically important during encoding but not recall (Linster et al., 2001; Linster and Cleland, 2002; Wilson et al., 2004, 2006; Mandairon et al., 2006; Chaudhury et al., 2009; Devore et al., 2014). We here show computationally that a feedback loop between olfactory cortex and the nucleus providing cholinergic input to the olfactory bulb and cortex, the HDB, allows the olfactory system to compare an odor stimulation to previously encoded odors and dynamically regulate if an odor will be encoded or not. The model presented relies on a projection, direct or indirect, between olfactory cortical pyramidal cells and HDB GABAergic interneurons. While such a connection has not been demonstrated, it has been shown that electrical stimulation of olfactory cortex can modulate neural activity in the HDB at a precise and short latency (Linster and Hasselmo, 2000), at least not excluding the possibility of such a projection. As a consequence, the possibility of feedback regulation exists in this system. Consequences of our proposed feedback loop are: (1) a relatively high spontaneous activity in cholinergic neurons in the HDB in the absence of odor stimulation, (2) a decrease in cholinergic neuron activity as an odor becomes increasingly familiar, accompanied by an increase in GABAergic activity in the HDB and (3) an increase in cortical pyramidal cell activity in response to increasingly familiar odorants. Our accompanying recordings from a small number of HDB neurons in a simple odor reward association task show that a small percentage of neurons each decreased or increased their firing rate significantly below or above their spontaneous rate over the course of sampling a same odor repeatedly. When presented with a second, novel odor, these neurons first return to their initial firing rate and then either decrease or increase their firing rates similarly as in response to the first odor. The return to baseline response levels upon initial presentation of the novel odor shows that the increase of decrease of activity is not merely a temporal phenomenon but depends on odor familiarity. Despite the small number of neurons recorded, the data are consistent in that all neurons clearly fell into three categories and hence we think can be representative for the behavior of HDB neurons in general for the particular behavioral task implemented here. While it is not possible to categorize GABAergic and cholinergic neurons from these recordings it cannot be excluded that populations of HDB neurons (GABAergic, cholinergic or glutamatergic) could correspond to the separate responses in this particular task. Overall, these recordings are in agreement with the predictions from our computational model as presented here; however the data presented is not exclusively compatible with the theoretical ideas presented here but could result from a number of different scenarios. For example, internal synaptic connections between HDB neurons, as evidence by Yang et al. (2014) could result in differential activities of HDB neurons as observed here.

Our simulations show that cortical learning could modulate olfactory bulb odor representations by modulating the degree of cholinergic inputs. As a consequence, encoded OB odor representations are sparse and synchronous, with high signal to noise ratio. As we have shown before (de Almeida et al., 2013; Devore et al., 2014), cholinergic modulation of OB processing enhances cortical learning and stability and duration of cortical representations. In the context presented here, cholinergic input to the OB is not strictly necessary for the regulation of learning to occur but enhances the specificity of the learned odor representation. In our simulations, the size of the cortical attractor decides if an odor is considered familiar or novel. Together with previous experimental and computational work from olfactory and other systems, our results strongly suggest the existence of a feedback loop between sensory representations and learning, mediated by cholinergic projections.

## Author contributions

LD designed, implemented, ran and analyzed computational modeling results and drafted manuscript. CL and MI designed and analyzed computational model. OD performed electrophysiology experiments. CL analyzed electrophysiological data and drafted manuscript. SD and DS designed experiments and edited manuscript.

### Conflict of interest statement

The authors declare that the research was conducted in the absence of any commercial or financial relationships that could be construed as a potential conflict of interest.
